# Dental caries - A complete changeover (Part I)

**DOI:** 10.4103/0972-0707.55617

**Published:** 2009

**Authors:** Carounanidy Usha, Sathyanarayanan R

**Affiliations:** Department of Dentistry, Pondicherry Institute of Medical Sciences, Pondicherry, India; 1 Department of Conservative Dentistry and Endodontics, Bapuji Dental College and Hospital, Davangere, Karnataka, India

**Keywords:** Dental caries, biofilm, dental plaque, demineralization, remineralization, ecological plaque hypothesis, oral fluid, sucrose, cariogenic, *S. mutans*, Lactobacilli

## Abstract

In spite of a knowledge explosion in cariology science, dental caries still remains a misunderstood phenomenon by the clinicians. In order to effectively use the wide range of preventive and management strategies, it is imperative to look beyond those black and white spots that manifest on the tooth surfaces. This paper focuses on the revised versions of the etiopathogenesis and definition of dental caries disease in the present century.

## INTRODUCTION

This serial review shall begin with happy conclusions; these are perhaps promising beginnings and not the end in cariology. They are as follows:

1. A global decline in dental caries in all age groups.
2. The altered perception of the profession to view dental caries as a ‘disease’ and the cavitation that is seen in the tooth, in whatever stage or color, as the manifestation of the disease—‘the lesion.’

## SUCCESSFUL DECLINE

Dental caries is as ancient as mankind and has the longest association with the dental profession, an association that is punctuated with agony and ecstasy. The agonizing fact is that despite several efforts towards total eradication, this disease is still prevalent. Nevertheless, an ecstatic success of the profession is the global decline in the incidence compared to the yesteryears' epidemics.[1] This success can be attributed to the exhaustive and extensive study of dental caries along with enhanced public awareness, advancements in dental material science, and the widespread availability of dental services.

## DISEASE *VS.* LESION

For many centuries, dental science remained happily divorced from biology and was very contented to be married to mechanics, despite the warning of Dr. G. V. Black against such an estrangement in 1908.[2] Caries in Latin means, ‘rotten’. For a nonprofessional, caries meant a hole in the tooth and for the dental professionals, it meant destruction of the tooth structure in the form of cavitation. We, ‘operative surgeons,’ logically adopted the surgical model of treatment for such a cavity, only to make a bigger, more geometrically perfect cavity, and fill it with the most compatible, artificial materials. This surgical model of ‘drill and fill’ resulted in more ‘drills and fills’ and the tooth seemed to land from the socket into the bucket, which Simonsen later called the ‘Molar cycle’.[3] The surgical model made us feel like Lilliputians desperately trying to contain the gigantic Gulliver.

Despite several breakthroughs in systematic scientific research in dental caries that started as early as the 1800s, this concept of making stereotypical, preconceived shapes of cavities prevailed in dental training programs as well. This is well stated by EAM Kidd “…as soft, brown, demineralized dentin only appeared in the clinic, as a considerable inconvenience, to view these preconceived shapes.”[4]

As the saying, ‘seeing is believing’ suggest, it was easier to believe in the effect that was seen (the cavity) and work on it and difficult or even impossible to imagine the unseen pathological causal mechanism (the disease) that resulted in this obvious effect. However, a paradigm shift is an inherent characteristic of any evolving science and it happened to cariology too. The shift was from the ‘mechanics-based surgical model to a biology-based medical model’ of disease management.

Any disease should have causative factor(s), pathogenesis, clinical manifestation or a sign, and predisposing risk factors. Dental caries perfectly fitted into this medical model of disease as background knowledge of this condition kept expanding. This shift paved the way for a double-pronged attack on the disease and its lesion.

The above two successful changeovers are not just stand-alone changeovers, but are associated with many other radical changeovers. Undoubtedly, this metamorphosis of dental caries may not be entirely new to the reader, but a comprehensive understanding of these changes and their consequences may still be lacking. Therefore, the need of the hour is to link all these transformations into one logical scientific chain and translate them for effective application.

## DISEASE ETIOLOGY–THEN AND NOW

It is inherent in human behavior to compulsively assess, analyze, and associate a cause with its effect. This has been the inevitable impetus for the progress of mankind. Dental caries is no exception to this compulsion and numerous theories were devised since the BC era. Many of them were refuted and negated, whereas some of them sustained the scrutiny of science. The reader is encouraged to refer to some exhaustive reviews by well-read authors.[5]

One such theory that has been accepted almost universally, but not without modifications, is the ‘chemo-parasitic theory.’This theory proposed by W. D. Miller in 1881[6] elucidated a combined effort of the acids (chemo) and the oral microorganisms (parasites) in tooth decalcification. Incidentally, this theory has evolved along with the revolutionary research of microbiologists such as Louis Pasteur and Robert Koch. According to this theory, the microorganisms in the oral cavity metabolize the dietary starch and produce organic acid that dissolves tooth minerals.

Miller's theory, in fact, had inadequacies in the explanation of the causation of dental caries, but it became an inevitable backbone for future studies in the discipline of cariology. They are as follows:

- According to this proposal, ‘all and any of the salivary microorganisms’ that were acidogenic (acid-producing) were responsible for the decalcification of the tooth structure. On the contrary, the elegant work of G. V. Black and J. L. Williamin 1898[7] explained the entity of ‘dental plaque,’ a colonization of endogenous microorganisms on the tooth surface that caused tooth dissolution.
- From the 1950s to 60s, the landmark studies of Orland *et al.* and the ‘Keyes and Fitzgerald revolution’[8] proved the strong causal relationship of certain specific microorganisms such as Streptococci, Lactobacilli, and Actinomyces present in the dental plaque with the incidence of caries. These studies also postulated that dental caries is a transmissible, microbial disease.
- Miller's other finding was that starch is more detrimental than sugar in terms of acid production. However, the significance of acid, its relationship to various sugars, and to varying times of contact with teeth have been proved by the famous Stephan's curve of 1940.[9] The pH drop with the intake of complex and simple sugars was plotted in a graph. Simple sugars, such as sucrose, tended to produce more acidity in a short period of time which lasted longer and took longer to come to neutrality when compared to complex starches. Even interventional human studies such as the famous Vipeholm study[10] conducted from 1945 to 1952 and the Hopewood study[11] in 1942 proved the definitive relationship of dietary sugars, the quantity, quality, and frequency of intake on the incidence and prevalence of caries. In the Vipeholm study, it was observed that the study's institutionalized patients experienced more caries when their diet was changed to a sugar-containing one. In the Hopewood study, it was reported that children taken into the Hopewood House in infancy did not develop any dental caries as long as they were provided food that did not contain refined sugar. However, they noticeably developed caries when they left the house and changed their diets to sugar-containing ones. The 1986 COMA report of UK[12] concluded, among other facts, that there is a positive relationship between caries and the consumption of non-milk extrinsic sugar (like table sugar and glucose syrup).

## CAUSE AND EFFECT MODELS—THEN AND NOW

Summing up all the refined observations made in the framework of Miller's theory, we find that all of them point accusing fingers at three major factors as causative factors in dental caries. Every one of us must be familiar with Venn's diagram with three intersecting circles. Two circles depict diet and dental plaque / microbial factors and third circle depicts the host. The plaque and the dietary factors are interdependent on each other for the causation of damage. The host factor becomes the ‘platform’ for this interaction, as well as the‘victim’ in the interaction of the other two factors. The intersected section of all these three circles depicts caries. Yet another circle was added not so very recently as the time / frequency factor. In other words, it depicts the duration of the interaction of the above factors. The longer the interaction of the dietary sucrose and the cariogenic microbes in the plaque, the more deleterious is the effect of acid on the dissolution of tooth mineral. This automatically indicates the frequency of sugar consumption by the individual.

**Figure 1:**
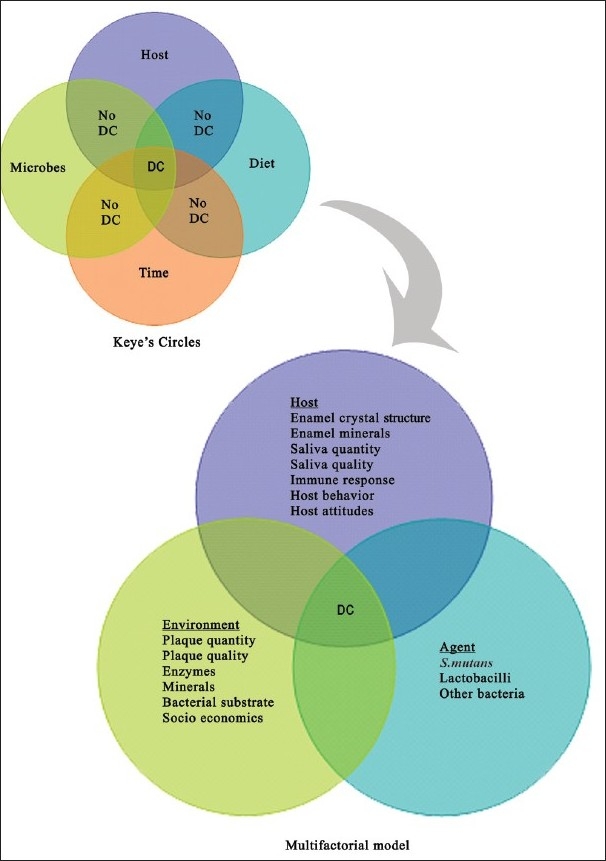
Cause and effect models

In order to make the model more specific, the major circles were modified to include the innumerable minor factors within the major factors.[13] Each of these factors has a precise role to play in the interplay of major factors, resulting in numerous permutations and combinations of the causative factors. It is very evident that this model encompasses almost all the key findings of various studies conducted along the timeline of cariology [Figure 1].

**Figure 2:**
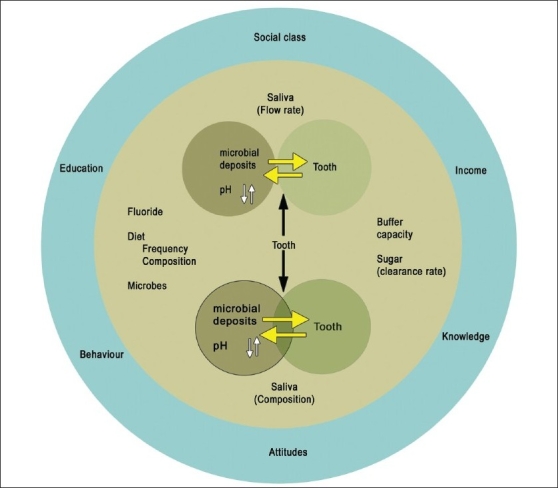
‘Determinants - Confounders’ model in dental caries

These models are, however, considered now to have oversimplified the complex behavior of the disease and are therefore, alleged to have contributed to the incomplete success of the management and prevention at the individual as well as the community levels. It is very common to encounter patients who have a ‘sweet tooth’ but do not suffer from caries, and also those who are very conscious of their sugar intake and yet have a high risk for the disease. This confounding fact is not explained by these models. On the contrary, they erroneously convey the message that all the factors have to be present at the same time with the same intensity to produce the disease. It is indeed true that multiple factors have to act in concert with each other to produce the disease, but not necessarily at the same time. To explain this inadequacy, the circles are simply rearranged in a different manner and the factors have been categorized as ‘determinants’ and ‘confounders’[14] [Figure 2].

A determinant is anything that determines the outcome. Most of the major and minor components are determinants because of the way(s)they interact with each other to determine the outcome-enamel dissolution. The variations in the risk status between two individuals with similar determinants as well as the variations in the determinant character between two individuals with similar risk status is explained by the components called confounders which are placed at the periphery.[15]

An interesting as well as debatable concept that is prevalent in the science of epidemiology is called ‘causal inference.’[16] Contributory factors are categorized as sufficient causes and / or necessary causes. For example, any cause that is ‘sufficient as well as necessary’ for the outcome is ‘the cause’ of the disease. In other words, this actor will always be present when the disease is present. Thus, a factor can also be either ‘a sufficient but not necessary cause’, or ‘not sufficient but a necessary cause’, or ‘neither sufficient nor necessary cause.’ Rothman's ‘sufficient cause’ concept has been recommended recently by Bokhout *et al*.,[17] as an ideal concept to assess the extent of the relationship between determinants and dental caries by using multivariate analysis. However, this concept was expertly analyzed and refuted earlier with regards to dental caries by Thylstrup and Fejerskov.[14]

## LESION MANIFESTATION—THEN AND NOW

Past definitions of dental caries always projected it as a progressive demineralization resulting in the destruction of the tooth structure. Even the latest definitions in certain textbooks mention dental caries as an irreversible disease.[18] However, as stated by Ernest Newbrun,[19] “Caries is not simply a continuous and unidirectional process of the demineralization of the mineral phase, but appears to be cyclic, with periods of demineralization immediately following metabolism of a fermentable substrate by the plaque flora, interspersed with periods of remineralization.”

Surprisingly, this concept was studied extensively as early as the1900s by several *in vitro* as well as *in vivo* models.[20] The research started with the search for the rationale behind the presence of arrested caries in certain individuals. It was even termed as ‘caries reversibility or consolidation.’[18] Only now, after the paradigm has shifted towards the medical model of dental caries, has remineralization gained more significance. It is encouraging to understand that there is a ‘default pause button’ in the disease progress, which can be taken advantage of in arresting the progress or even preventing the disease.

## THE PATHOGENESIS—THEN AND NOW

The oral cavity houses more than 250 microbial species. Unlike oral epithelium, the epithelium of the tooth does not shed and tooth morphology has many areas inaccessible to physiological clearance mechanisms. Thus, a tooth becomes an ideal place for the stubborn adherence for many of these species. This colonization occurs as a string of methodical adhesion, succession, and progression.[21] Organisms that are capable of adhesion adhere to the salivary pellicle on the tooth and form a convenient arena for the subsequent aggregation of other organisms that are incapable of initial adhesion. These are all endogenous / host microorganisms, not external infectious agents. In fact, this natural colonization of the tooth prevents any invasion by an exogenous organism by way of colonization resistance.

**Figure 3:**
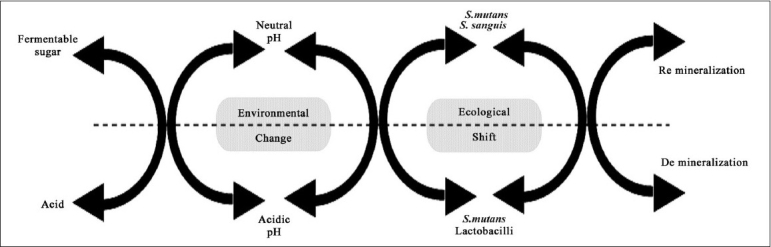
Ecological plaque hypothesis

For many years, either all plaque flora were collectively considered as being pathogenic (nonspecific plaque hypothesis—NSPH) or certain specific organisms were considered pathogenic (specific Plaque hypothesis—SPH).[22] Under NSPH, the target was to remove the entire plaque, but it was slowly realized that it was impossible to remove this natural accumulation of microbes on the tooth, even after tooth brushing or professional cleaning. Under SPH, the target was to eliminate specific pathogens with antimicrobial treatment. This hypothesis failed to substantiate the inability to detect these specific organisms in the presence of the disease or vice versa. In 1991, a new hypothesis was proposed called the ‘ecological plaque hypothesis’[23] [Figure 3]. According to this hypothesis, a certain change in the environment of the residential plaque flora provides pathogenicity to specific species that produce the disease only at specific sites. The ecological plaque hypothesis not only targeted specific species, but also targeted the factors that resulted in the environmental change of the plaque. A detailed explanation of this hypothesis will follow shortly.

The pathogenesis of dental caries in the present scenario will be discussed in the following section as disturbances in two types of homeostasis / physiological equilibrium that exist in an oral cavity. They are not independent disturbances and for the sake of discussion, they are categorized as follows:

1. Disruption of microbial homeostasis in the ‘biofilm’.
2. Disruption of mineral homeostasis between the tooth and the ‘oral fluid’.

It is imperative to understand the newly introduced terms, ‘biofilm’ and ‘oral fluid’ before proceeding further.

A biofilm is defined as the population or community of bacteria living in organized structures at an interface between a solid and liquid. A closer study of plaque reveals a much more organized structure than what the traditional definition of plaque depicts. Various microbial species are found to occupy their respective microcosms that are unique to their survival, with interspersed channels that segregate them. These channels act like a two-way transport for the nutrition and byproducts of the microbes. Therefore, the term, ‘biofilm’ is considered more apt terminology than plaque.[24] Oral fluid encompasses the saliva and the gingival crevicular fluid. These fluids actively contribute to the onset or prevention of the disease by virtue of their contents and functions.

## DISTURBANCE IN MICROBIAL HOMEOSTASIS

Various species live in a co-operative, physiological equilibrium in biofilm through a complex interaction of synergism and antagonism. The microbial colony in the tooth can sustain mild disturbances in its ambience without much alteration in its structure and composition. Such disturbances are usually abundant in a dynamic oral cavity. Nevertheless, if the disturbance is severe and persistent, it results in the shift in the equilibrium. The organisms that cannot survive in such adverse environs perish and those that can, flourish. Thus, the resulting change in the structure and composition renders the host plaque hostile.

One of the initial colonizers on the tooth surface is the Streptococcus species, predominantly, *S. oralis*, *S. sanguis*, and *S. mitis* with *S. mutans* comprising only 1% of the colonizing population.[25] Flora in the biofilm can metabolize fermentable simple sugars in the diet through complex biochemical pathways to produce various organic acids as the byproducts, resulting in enamel dissolution. However, oral fluids buffer the acidic interface within 30–60 minutes with bicarbonates and phosphates. This kind of fluctuation in pH occurs with every attack of sugar on the biofilm. Only if the frequency / intensity of the acid attack outweigh the buffering capacity of the oral fluid will the physiologic equilibrium in the microbial colony change. The continuous acidic environment allows only the aciduric (acid-resistant) organisms such as the *S. mutans* and lactobacilli to proliferate in larger numbers. As they are acidogenic also, the local acid production keeps increasing. Thus, *S. mutans* and lactobacilli that were initially low on the list of colonizers later predominate in a mature, cariogenic biofilm. These are homofermentative in nature, which means that they produce mainly lactic acid instead of the other organic acids.

**Figure 4:**
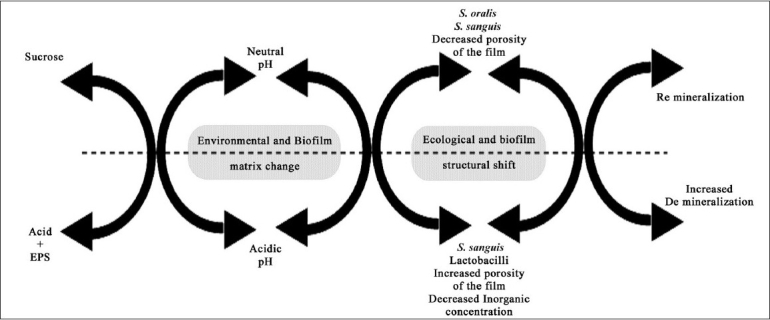
Influence of sucrose on the environmental change

The role of dietary sucrose deserves a mention here [Figure 4]. The cariogenicity of sucrose is attributed to its easy fermentability compared to other starches. It also provokes *S. mutans* to synthesize ‘extracellular (EPS) and intracellular polysaccharides (IPS).’ Extracellular polysaccharides (glucans) enhance the bacterial adherence, improve the structural integrity, and increase the bulk of the biofilm. Intracellular polysaccharides act like a storage medium for the sugar inside the bacterial cell to be metabolized during nutrient starvation periods, thus prolonging the acidity around the tooth, even in the absence of dietary sugar.[26]

## DISTURBANCE IN MINERAL HOMEOSTASIS

**Figure 5:**
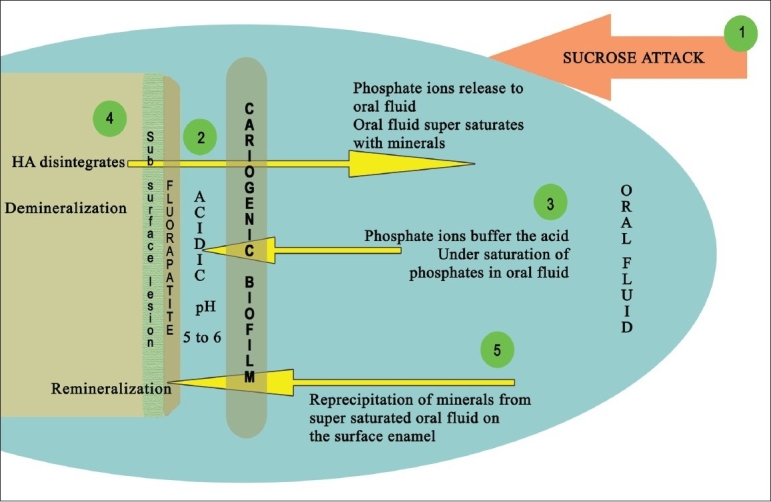
Demineralization of hydroxyapatite (HA) and remineralization with fluorapatite (FA) - depicted as stage 1–5 Stage 1: Fermentable sucrose intake. Stage 2: Microbes in cariogenic plaque metabolise them releasing acid in the biofilm-tooth interface. The pH in the interface drops below the critical pH of HA Stage 3: Phosphate ions from oral fluid buffer the acidic ions resulting in undersaturation. Stage 4: HA disintegrates to release the phosphate ions back to the oral fluid till it supersaturates-Demineralization Stage 5: Supersaturated oral fluid re precipitates the minerals onto the disintegrated enamel. If fluoride also deposits FA is formed on the superficial layer - Remineralization. Sub surface demineralization remains.

Tooth enamel is composed primarily of hydroxyapatite (HA)—Ca_10_(PO_4_)_6_(OH)_2_ [Figure 5]. Oral fluid is a rich reservoir of calcium, phosphate, and fluoride minerals. According to the law of saturation, a dynamic equilibrium exists between the mineral contents of the tooth and the oral fluid[27] with the mineral content in the HA crystal being equal to that of the oral fluid.

In any aqueous ambience with a ‘neutral pH,’ the HA crystal dissolves minimally and releases the calcium (Ca^2+^), phosphate (PO_4_^3−^) and hydroxyl (OH−) ions into the solution. If the solution, for instance, is the oral fluid that already contains the same minerals, it becomes ‘supersaturated’, resulting in the ‘precipitation’ of the minerals back onto the tooth enamel.

In an aqueous ambience with ‘acidic pH,’ mainly the phosphate ions, (PO_4_)^3−^, and the hydroxyl ions (OH−) react with the hydrogen ions (H^+^) in the tooth–biofilm interface, by forming complexes, such as, HPO_4_^2−^ and H_2_0. In a severe acidic environment hydrogen ions (H^+^) further combine with HPO_4_^2−^, to form H_2_PO_4_^2−^, a more acidic ion.[28]

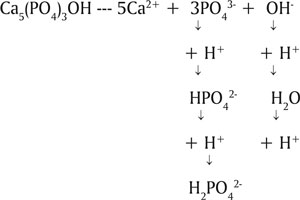



Thus the localized acidity is buffered but the oral fluid becomes ‘undersaturated’ with respect to the phosphate ions (PO_4_^ 3−^). This leads to the ‘dissolution’ of HA crystals in an attempt to resaturate the oral fluid. This dissolution reaction is described as follows:

${\mathrm{Ca}}_{10}{\left({\mathrm{PO}}_{4}\right)}_{6}{\left(\mathrm{OH}\right)}_{2}+14H^{+}\to 10{\mathrm{Ca}}^{2+}+6H_{2}{\mathrm{PO}}_{4}^{-}+H_{2}O$ [29]

Thus, it is evident that the dissolution of the HA crystals occurs at a certain acidic pH, which is referred as being ‘below the critical pH of HA.’ The ‘critical pH’ is the pH at which a solution is just saturated with respect to a particular mineral. For the HA crystal, it is approximately 5.5–6, below which the enamel disintegrates.

This is a very simplified explanation for a complex process of the mineral's dynamic equilibrium as well as the demineralization / remineralization balance.

The chemical reactions of de- and remineralization are also explained in relation to the ‘ion activity product’ (IAP in the solution) vs. ‘*K_sp_’* (Solubility product constant of enamel)’.[28,29] The IAP denotes the product of the activity of the ions released into the solution during dissolution and is expressed with regards to HA crystals as (Ca^2+^)^10^ × (PO_4_^3−^)^6^ × (OH^−^)^2^. The K_sp_ for HA at 37 degree C is 7.41 × 10^−60^mmol^9^/l^9^. The solution is said to be in equilibrium with HA when IAP = K_sp_. Demineralization occurs when IAP in the solution < K_sp_, and remineralization occurs when IAP in the solution > K_sp_.

Remineralized primary enamel (HA with soluble impurities such as fluorides and carbonates) is rendered acid-resistant to a considerable extent in this dynamic balance. If the oral fluid contains fluoride along with calcium and phosphate, then a much more acid-resistant structure develops on the superficial layer of the enamel and is called, ‘fluorapatite’ (FA).

The critical pH of FA crystals is around 4.5 so that FA can demineralize only when pH < 4.5 (greater acidity). This can occur if the biofilm's cariogenicity is intense, the sucrose attack is persistent, and the protective functions of the oral fluid, such as flow, quantity, and quality, are compromised.[27] The pH in the deepest portion of a thick biofilm that is not amenable to oral hygiene measures, is more acidic compared to saliva. This is attributed to the inability of the oral fluid to penetrate the thick biomass to exert its buffering capacity, as well as to the altered microbial content (more acidogenic and aciduric organisms) here. The strict adherence of *S. mutans* to the tooth through its extracellular polysaccharides further intensifies the situation. It is also evident from earlier studies that the calcium and phosphate contents in biofilm fluid are lower than the salivary contents.[26] This means that the biofilm fluid is always undersaturated with mineral ions with respect to HA / FA crystals. This results in a continuous loss of mineral from the enamel layer, which can culminate in the devastating ‘cavitation.’

It is interesting to note that the entire demineralization and remineralization process is limited to only few millimeters of superficial enamel. The ‘subsurface’ enamel is always in a demineralized state with a minimum of 0.03 mm of intact surface enamel. The reason for this is the inadequate penetration of the mineral ions during precipitation. What sounds like a handicap in Nature's process is in fact a boon with respect to the non-interventional management of such a ‘white, opaque, incipient lesion.’ Here begins the revolution in lesion detection and management.

The role of saliva in oral fluid is obvious in this entire process. The quantity, quality, buffering capacity, and the composition of saliva determine the dissolution and precipitation of HA. In addition, the salivary pellicle plays a very significant role in the protection of the tooth surface from acid attack. It also prevents excessive mineral deposition on the tooth from a supersaturated oral fluid.

Thus, etiological factors are broadly categorized as, ‘demineralizing / pathological factors’ and ‘remineralizing / protective factors.’[30] Demineralizing factors are enlisted below:

- Fermentable sucrose-containing diet
- Cariogenic biofilm with *S. mutans* and lactobacilli
- Acidic by products
- Low salivary flow
- Low buffering capacity of both saliva and the biofilm fluid
- Reduced oral clearance rate
- Inadequate mineral content of the saliva and the biofilm

It is quite easy to understand that remineralizing factors are almost the opposite of the above list.

## THE END AND THE BEGINNING

It is strongly recommended that readers keep the links intact between the above sections, although they have been individually subtitled for the sake of easy reading. None of these event(s) act independently in the causation of dental caries.

To sum up, the revised etiopathogenesis models and remineralization concepts have given a forward thrust to the science of cariology as follows:

1. It became imperative to detect these incipient lesions at the earliest to take advantage of the inherent capacity of the body to heal by itself. Thus, detection incorporated sophistication to the point of precision.
2. It broadened our minds while narrowing our vision to identify the modified, ‘miniaturized’ presentation of the dental carious lesions.
3. The need to change the traditional classification was realized so as to be abreast with the newer perspectives.
4. It paved a logical route towards minimal sacrifice and maximal preservation of the natural tooth substance.
5. The possibility of producing caries-free generation emerged with the concepts of immunization against caries.
